# CPAP Therapy Termination Rates by OSA Phenotype: A French Nationwide Database Analysis

**DOI:** 10.3390/jcm10050936

**Published:** 2021-03-01

**Authors:** Jean-Louis Pépin, Sébastien Bailly, Pierre Rinder, Dan Adler, Daniel Szeftel, Atul Malhotra, Peter A. Cistulli, Adam Benjafield, Florent Lavergne, Anne Josseran, Renaud Tamisier, Pierre Hornus, on behalf of the medXcloud Group

**Affiliations:** 1 HP2 Laboratory, University Grenoble Alpes, Inserm, CHU Grenoble Alpes, 38043 Grenoble, France; Sbailly@chu-grenoble.fr (S.B.); rtamisier@chu-grenoble.fr (R.T.); 2 SEMEIA, 75010 Paris, France; prinder@semeia.io (P.R.); dszeftel@semeia.io (D.S.); phornus@semeia.io (P.H.); 3 Faculty of Medicine, University of Geneva, 1205 Geneva, Switzerland; dan.adler@hcuge.ch; 4 Pulmonary, Critical Care and Sleep Medicine, University of California, San Diego, CA 92037, USA; amalhotra@health.ucsd.edu; 5 Charles Perkins Centre, Faculty of Medicine and Health, University Sydney, Sydney, NSW 2006, Australia; peter.cistulli@sydney.edu.au; 6 ResMed Science Center, Sydney, NSW 2153, Australia; Adam.Benjafield@resmed.com.au; 7 ResMed Science Center, 69791 Saint Priest, France; florent.lavergne@resmed.fr (F.L.); anne.josseran@resmed.fr (A.J.)

**Keywords:** obstructive sleep apnea, continuous positive airway pressure, comorbidities, adherence, treatment

## Abstract

The nationwide claims data lake for sleep apnoea (ALASKA)—real-life data for understanding and increasing obstructive sleep apnea (OSA) quality of care study—investigated long-term continuous positive airway pressure (CPAP) termination rates, focusing on the contribution of comorbidities. The French national health insurance reimbursement system data for new CPAP users aged ≥18 years were analyzed. Innovative algorithms were used to determine the presence of specific comorbidities (hypertension, diabetes and chronic obstructive pulmonary disease (COPD)). Therapy termination was defined as cessation of CPAP reimbursements. A total of 480,000 patients were included (mean age 59.3 ± 13.6 years, 65.4% male). An amount of 50.7, 24.4 and 4.3% of patients, respectively, had hypertension, diabetes and COPD. Overall CPAP termination rates after 1, 2 and 3 years were 23.1, 37.1 and 47.7%, respectively. On multivariable analysis, age categories, female sex (1.09 (1.08–1.10) and COPD (1.12 (1.10–1.13)) and diabetes (1.18 (1.16–1.19)) were significantly associated with higher CPAP termination risk; patients with hypertension were more likely to continue using CPAP (hazard ratio 0.96 (95% confidence interval 0.95–0.97)). Therapy termination rates were highest in younger or older patients with ≥1 comorbidity. Comorbidities have an important influence on long-term CPAP continuation in patients with OSA.

## 1. Introduction

Obstructive sleep apnea (OSA) is a highly prevalent chronic respiratory condition. Global estimates suggest that almost 1 billion people aged 30–69 years have OSA (defined as an apnea–hypopnea index (AHI >5/h)); of these, 425 million have moderate to severe disease (AHI ≥15/h) [1]. A substantial body of evidence suggests that the presence of OSA contributes to a number of poor health outcomes, including neurocognitive impairment, hypertension and cardiovascular disease and early mortality [2,3].

In the USA alone, the cost of diagnosing and treating OSA in 2015 was approximately USD 12.4 billion, approximately half of which was attributed to the cost of treatments including positive airway pressure and oral appliance therapy [4]. However, the cost of undiagnosed OSA was even higher (USD 149.6 billion) as a result of lost productivity and absenteeism, medical comorbidities and workplace and motor vehicle accidents [4]. Therefore, the diagnosis and treatment of OSA is increasingly being recognized as an important component of the long-term management of chronic, noncommunicable diseases [5].

Although intermittent episodes of upper airway obstruction during sleep are needed for diagnosis and are a common feature among all patients with OSA, disease presentation, symptoms and comorbidities including obesity differ between patients [6,7]. Some patients have few or no symptoms, while others experience symptoms such as excessive daytime sleepiness; OSA may be present alone or might be accompanied by one or more comorbidities, and the cardiovascular risk profile varies between patients. This situation results in a number of different OSA phenotypes with differing treatment requirements and goals [8].

The gold standard first-line treatment for OSA is continuous positive airway pressure (CPAP) and is used by 1.2 million individuals in France. One of the major issues with long-term CPAP therapy is adherence. Clinical trial data show that fewer than 50% of patients are using CPAP for >4 h/night after 1 year [9], but this threshold of device usage may be essential for the benefits of therapy to be realized [10,11]. Disease severity, psychological factors and management of side effects related to CPAP have been associated with treatment adherence [12]. Moreover, it has recently been reported that physical and social characteristics of the patient–spouse couple also impact on adherence [13]. This finding highlights the possibility that other patient characteristics could contribute to long-term adherence to CPAP therapy. However, there is a lack of robust data on the role that different comorbidities might play in the persistence with, or termination of, CPAP therapy.

The nationwide claims data lake for sleep apnoea (ALASKA), real life data for understanding and increasing OSA quality of care study, investigated 1- to 3-year CPAP therapy termination rates in France using data from the national health insurance reimbursement system database and determined whether patients with specific comorbidities (hypertension, type 1 or type 2 diabetes and chronic obstructive pulmonary disease) were at higher risk for CPAP therapy termination. 

## 2. Methods

### 2.1. French National Claims

This cohort analysis included data from the French national health insurance reimbursement system database (“Système national des données de santé”; SNDS), which contains individualized, anonymous and comprehensive data on health spending reimbursements and covers >99% of all individuals living in France. The ALASKA project has been approved by the “Commission Nationale Informatique et Liberté” (CNIL), the French information technology and personal data protection authority. Specific approval was obtained from the CNIL to perform this study (DR-2019-78 and n°919194). 

### 2.2. Study Population and Definition of CPAP Therapy Termination

The study cohort included adults aged ≥18 years who were naive users starting CPAP therapy during the period from January 2015 to December 2016; CPAP use and a diagnosis of OSA were identified by specific treatment modality and disease codes, respectively. Patients in France are eligible to have CPAP prescribed if they have at least three clinical symptoms (daytime sleepiness, severe and daily snoring, sensations of choking during sleep, daytime fatigue, nocturia and/or morning headaches) and meet one of the following criteria: AHI ≥30/h based on total sleep time (TST) during polygraphy (PG) or polysomnography (PSG); AHI ≥15/h and <30/h based on TST during PG or PSG with severe daytime sleepiness and/or accidental risk causing direct or indirect injury; AHI ≥15/h and <30/h based on TST during PG or PSG and severe cardiovascular or respiratory comorbidity (resistant hypertension, atrial fibrillation, symptomatic heart failure, coronary artery disease, history of stroke, severe COPD or poorly controlled asthma). 

CPAP therapy termination was defined as the cessation of CPAP reimbursements triggered by the respiratory physician or sleep specialist in charge of follow up. French national recommendations for reimbursement are CPAP usage of >4 h/night, and reimbursement rates progressively decrease when there is very low adherence to CPAP. However, CPAP delivery and reimbursement can continue when adherence is between 2 and 4 h/night pending additional patient education and coaching. Ultimately, the decision to terminate therapy was made by the treating physician taking into account adherence as well as perceived benefit from the perspective of both the patient and physician. Mandatory follow-up visits are required at 4 months after CPAP initiation and every year for treatment reimbursement renewal. CPAP termination was considered to be linked to non-adherence and included in the Kaplan–Meier analysis, or to be associated with a valid explanation (sleep apnea cure after bariatric surgery, otorhinolaryngology surgery, switch to oral appliances, death). Where there was one of these valid explanations for CPAP termination, the individual was censored in the Kaplan–Meier analysis.

### 2.3. Methodology for Comorbidity Identification in SNDS

SEMEIA collaborators (P.H., P.R. and D.S.) developed specific algorithms to improve comorbidity identification in the SNDS database. SNDS anonymously links information for all health care insurance reimbursement claims to the national hospital discharge database (PMSI). The comorbidities of interest in the current analysis were hypertension, diabetes and chronic obstructive pulmonary disease (COPD). Identification of these comorbidities was performed using a combination of four complementary approaches: (1) Information is available and coded based on the ICD-10 code for severe and costly chronic diseases (affection de longue durée (ALD)) reimbursed at a 100% rate in France. (2) Detection of comorbidities using ICD codes (full list available in the online supplement). The PMSI database provides detailed medical information about all stays in public and private hospitals, including main and associated discharge diagnosis ICD-10 codes. (3) Identification was improved by searching consumption of specific comorbidity-related medications using ATC codes (full list available in the online supplement, Supplementary Tables S1–S6). To strengthen the recognition of comorbidities, medications related to comorbidities needed to have been delivered by both private and hospital pharmacies at least three times in the year preceding CPAP initiation. (4) Hospital stay for one of the comorbidities with corresponding ICD-10 code(s). All hospitalizations related to comorbidity-related complications were also identified using ICD-10 codes and were considered to identify comorbidities.

### 2.4. Statistical Analysis

Data are presented using descriptive statistics (mean and standard deviation for quantitative variables and frequency and percentage for qualitative variables). The primary dependent variable of interest was CPAP therapy termination. Primary independent variables included age, sex and comorbidities. In a first step, we compared rates of CPAP therapy termination according to age groups, sex and comorbidities using Kaplan–Meier survival analysis with log-rank test. Due to the large sample size, hazard proportionality assumption was not checked, and hazard ratio (HR) values must be interpreted as average HR rather than instantaneous HR [14].

The second step generated multivariate Cox proportional hazard models that included age, sex and comorbidities. Statistical significance was defined as a *p*-value < 0.05. Analyses were performed using Python 3.6.7 software (Python Software Foundation, Fredericksburg, VA, USA) with the libraries Numpy 1.18.1 and Pandas 0.24.2 for data management and analysis, and Lifelines 0.14.1 for Kaplan–Meier curves and Cox models.

## 3. Results

### 3.1. Study Population

A total of 480,000 patients were included in the analysis. The population was middle-aged (mean age 59.3 ± 13.6 years) and predominantly male (65.4%), which is typical of patients with severe OSA referred for CPAP initiation. The burden of the comorbidities of interest in this study was high, with hypertension, diabetes and COPD present in 50.7, 24.4 and 4.3% of patients, respectively.

### 3.2. CPAP Therapy Termination

**Figure 1:**
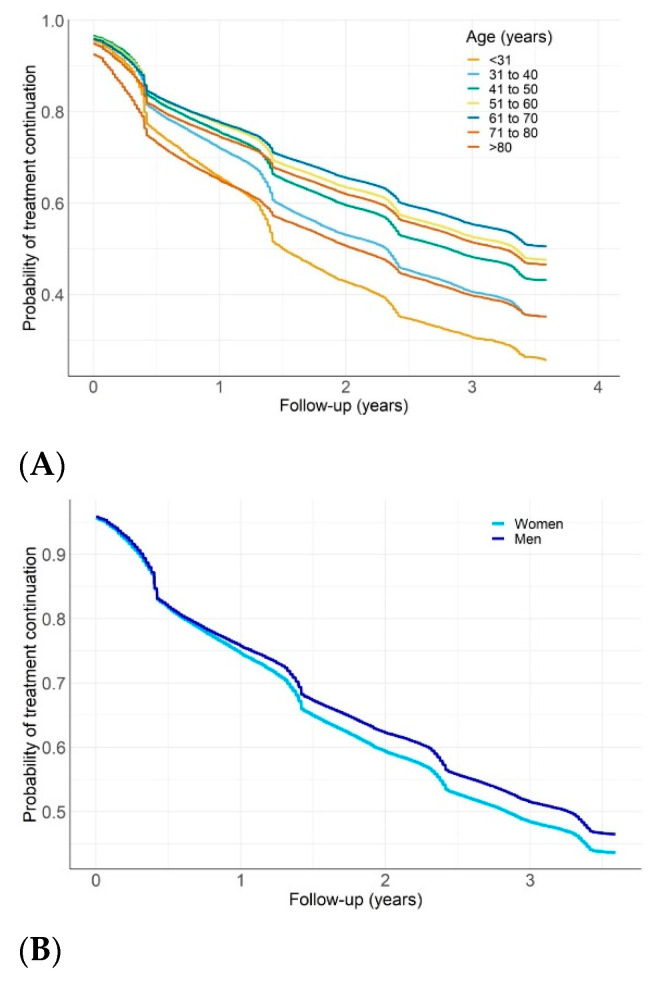

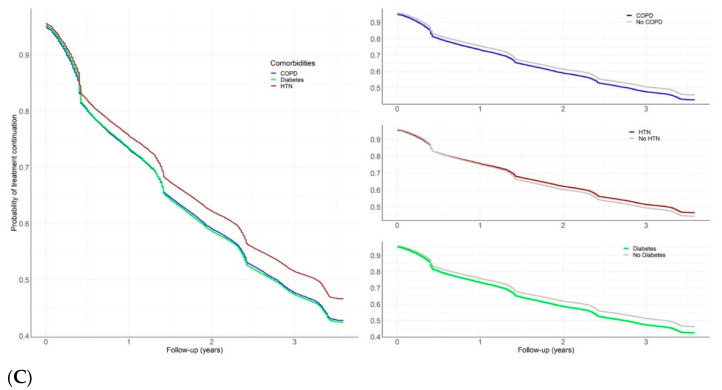
Probability of continuing continuous positive airway pressure (CPAP) therapy during follow-up in patient subgroups: (**A**) by age group; (**B**) by sex; (**C**) by comorbidities. COPD, chronic obstructive pulmonary disease; HTN, hypertension (log-rank test *p* < 0.01 for all comparisons).

Overall rates of CPAP therapy termination after 1, 2 and 3 years were 23.1, 37.1 and 47.7%, respectively. The proportion of patients terminating CPAP therapy for valid reasons was 1.7% (8192/480,000) (Supplementary Table S7). In unadjusted Kaplan–Meier survival analyses (Figure 1), younger patients (age 18–30 or 31–40 years), very elderly patients (age > 80 years) and females had higher rates of CPAP termination than patients in other age groups or males (log-rank *p* < 0.01). Termination rates were also higher in the presence of diabetes or COPD, but comparatively lower in patients with comorbid hypertension (log-rank *p* < 0.01, Figure 1). For patients with COPD, those who had an exacerbation-related hospitalization were significantly more likely to terminate CPAP than those who were not hospitalized (HR 1.21 (1.13; 1.29) versus 1.11 (1.09; 1.13), *p* < 0.01) (Supplementary Figure S1). 

**Figure 2:**
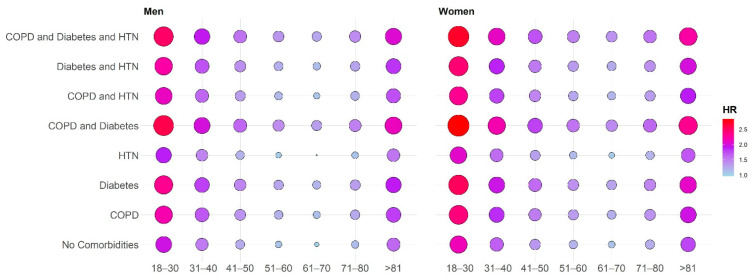
Balloon graph showing the risk of therapy termination in patient subgroups with different age group (years) and comorbidity combinations. COPD, chronic obstructive pulmonary disease; HR, hazard ratio; HTN, hypertension. The HR values reported in the balloon plot represent a graphical summary of the multivariable Cox model adjusted for age, sex and comorbidities. The reference patient in the multivariable model is a male patient without comorbidities aged 61–70 years. For the Cox model, the HR values are multiplicative after exponential transformation, so by multiplying the different HR values in the model, it is possible to depict the HR values for specific demographic and clinical scenarios.

In a multivariate Cox proportional hazard model, age 18–30 years (adjusted hazard ratio (95% confidence interval) 1.97 (1.91–2.03)), age 31–30 years (1.52 (1.49–1.55)), age 41–50 years (1.24 (1.22–1.26), age 51–60 years (1.08 (1.07–1.10)), age 71–80 years (1.13 (1.11–1.15)) and age >80 years (1.44 (1.41–1.47)) compared to age 61–70 years (reference), female sex (1.09 (1.08–1.10) and the presence of COPD (1.26 (1.23–1.29)) and diabetes (1.10 (1.09–1.11)) remained significantly associated with a higher risk of CPAP therapy termination (log-rank *p* < 0.0001). Patients with hypertension were more likely to continue using CPAP (hazard ratio 0.95 (95% confidence interval 0.94–0.96); log-rank *p* < 0.0001). The risk of therapy termination was highest in younger or older patients with one or more comorbidities (Figure 2, Supplementary Figure S2). 

## 4. Discussion

This study is the first to our knowledge to investigate the relation between a comorbid phenotype and the risk of CPAP therapy termination. The results showed that the overall rate of CPAP therapy termination for patients treated in the French health system was 48% at 3 years. In addition to age and gender, there was a clear and significant association between the presence of selected comorbidities and the risk of terminating CPAP therapy. The combination of young age, female sex and comorbid diabetes or COPD was most closely associated with CPAP therapy termination. 

Our findings of higher rates of therapy termination in females versus males, and in the youngest and oldest patient groups, are consistent with a big data analysis from Germany [15]. However, despite increasing recognition of OSA as an important contributor to chronic diseases [16], there has been little analysis to date regarding the effects of comorbidities on CPAP usage and therapy termination. Data from a prospective cohort study of 295 patients with moderate or severe OSA found a significant negative association between a history of cardiovascular events and CPAP adherence [9]. Other studies have also shown that adherence to CPAP decreases progressively over time in patients with concomitant OSA and cardiovascular disease [17,18]. Another recent database analysis investigated the role of comorbidities in predicting CPAP adherence [19]. Wickwire and colleagues used a 5% sample of Medicare claims data and determined adherence over a 13-month period for this population of older adults. Medical comorbidities significantly associated with reduced CPAP adherence were anemia and fibromyalgia [19]. Together with our study findings, these currently available data highlight the influence of variety of comorbidities on CPAP adherence and therapy termination.

There are a number of potential explanations for an association between the presence of diabetes and COPD in patients with OSA and high rates of therapy termination, as seen in our analysis. Firstly, patients with OSA and comorbidities often have no or few OSA symptoms [20,21], which could contribute to a higher rate of CPAP nonadherence. When patients with OSA were classified into three distinct clusters, those in the “minimally symptomatic” group had the highest probability of having comorbid hypertension and cardiovascular disease [22]. Suboptimal rates of adherence were documented in randomized clinical trials of CPAP in minimally symptomatic or asymptomatic patients with OSA and cardiovascular disease [10,11,23]. It has been suggested that low levels of adherence contributed to the lack of effect of CPAP on cardiovascular event endpoints in the intention-to-treat analyses of these studies [24]. In a study looking at response to positive airway pressure (PAP) therapy in patients with three different defined phenotypes of OSA (sleepy, minimally symptomatic and disturbed sleep groups), PAP usage was significantly higher in sleepy phenotype patients compared with the other groups (*p* = 0.034) [25]. Interestingly, our analysis showed that the presence of hypertension as a comorbidity was significantly associated with a greater likelihood of continuing with PAP therapy. This may be because physicians (and some patients) are aware of the BP-lowering effects of CPAP that have been documented in patients with hypertension or because the patients were more symptomatic [26], although the reasons underlying our finding warrant additional investigation.

In patients with comorbidities, the presence of OSA might contribute to difficulty in achieving control of the underlying condition (e.g., hypertension (blood pressure) or diabetes mellitus (blood glucose levels)) rather than resulting in typical OSA symptoms such as daytime sleepiness. For example, lack of daytime sleepiness in patients with heart failure and OSA is thought to be due to sympathetic overactivity [21]. Given the lack of symptoms, patients may not perceive OSA as having a detrimental effect on their health. Instead, they are likely to believe that their other conditions, such as diabetes or COPD, will have a greater impact on wellbeing and prognosis. These factors could contribute to lower prioritization of OSA and its treatment and could potentially be another explanation for the higher CPAP termination rates seen in patients with multiple comorbidities. This explanation is consistent with findings suggesting that patients’ perception of the benefit they obtain from CPAP therapy is a predictor of continued device usage [27,28]. Furthermore, the fact that CPAP has not had any significant effects on the cardiovascular event rate in randomized clinical trials to date [10,11,23] means that the benefits of this treatment approach in unselected patient groups may be difficult to justify [29]. In addition, both patients and their partners may have negative perceptions and experiences of CPAP therapy [30]. Therefore, patients without OSA symptoms could see CPAP as having a high burden for limited perceived benefit.

The inclusion of patients with a variety of different phenotypes in the clinical trials may have contributed to the overall lack of benefit of CPAP in the study populations. However, specific subsets of patients may have derived benefit from therapy. Zinchuk and colleagues used polysomnographic data to identify seven different patient clusters within subgroups based on traditional AHI cut-off values [31]. OSA disease severity based on the AHI was not significantly associated with cardiovascular risk, but several clusters showed significantly higher rates of cardio- and cerebrovascular events [31]. Consistent with our findings, CPAP usage was lowest in patients with comorbidities and in less sleepy patients. Furthermore, the ability of CPAP to reduce cardiovascular risk also varied between the different patient clusters [31]. From a clinical perspective, phenotyping of patients with OSA might allow more accurate determination of the potential for adherence to CPAP and for the benefits of therapy in specific patient groups, facilitating personalized medicine approaches to OSA management [32]. From a research perspective, phenotyping OSA could allow enrichment of clinical trial datasets with patients more likely to adhere to long-term treatment. CPAP is still a suitable approach for OSA patients with comorbidities, provided that appropriate management strategies are implemented. For example, OSA patients with a phenotype that may be associated with suboptimal adherence could be ideal candidates for the variety of digital tools now available to improve awareness, adherence and patient engagement with their PAP therapy [15,33,34]. Alternatively, some patients and patient groups may be more suited to the use of different approaches to treating OSA, such as mandibular advancement devices. 

A key strength of this study is that it uses a large exhaustive unbiased national dataset (covering >99% of the French population). This approach is in contrast to previous big data analyses conducted in the US and Europe that only included a subset of the population, such as those with a specific insurer or healthcare provider [15,33]. The French database can also be used to look at relationships between CPAP termination and worsening of comorbidities, because it contains data on parameters such as healthcare resource use and hospitalization, and these will be the subject of additional analyses. It may also be possible to link SNDS records with CPAP telemonitoring data to investigate additional relationships and predictors of therapy termination. Another unique feature of the study is the novel algorithm that was developed to detect comorbidities. Using specific codes, we have excluded patients requiring noninvasive ventilation for chronic respiratory failure or adaptive servo-ventilation for central sleep apnea. However, a subgroup of OSA with associated non-apneic sleep breathing disorders secondary to underlying diseases (e.g., asthma, interstitial lung diseases, neuromyopathies, chest wall and diaphragm diseases, obesity-hypoventilation syndrome) and/or non-respiratory sleep disorders (insomnia, restless leg syndrome) were not specifically excluded from analysis. These associated conditions may have influenced adherence to CPAP and need to be evaluated in future studies.

Despite having a number of strengths, there are also some limitations that need to be taken into account when interpreting the results of this study. Similar to other big data analyses [15,33], data were obtained from a database designed for administrative rather than research purposes. This means that a limited set of data is available. For example, there is no information on the apnea–hypopnea index, and therefore, OSA severity is unknown. In addition, the SNDS does not contain data on smoking habits, alcohol intake, body mass index and socioeconomic status. Another important limitation is that CPAP usage is a binary parameter (yes or no), and there is no information on the hours that the device is used each night. Therefore, it is possible that patients who did not terminate CPAP were not actually adhering to therapy in terms of optimal levels of device usage (traditionally defined as >4 h/night). Finally, this analysis only included a selected group of comorbidities (hypertension, diabetes and COPD). These were chosen arbitrarily because they are common and important comorbidities in patients with OSA, and because cardiorespiratory comorbidities are specifically mentioned in the French CPAP prescription rules. However, the effect of other cardiovascular diseases and other important comorbidities (e.g., mental health disorders, such as depression and anxiety, and other neurological diseases) on CPAP usage will be investigated in future studies. This is important due to the high prevalence of cardiovascular disease, and because psychological and neurological conditions have previously been shown to influence willingness to try CPAP therapy [35] and adherence to treatment [19].

In conclusion, this analysis of a dataset covering almost the entire French population showed that the presence of comorbidities was an important contributor to termination or continuation of CPAP therapy in patients with OSA. Given the diversity of OSA patient phenotypes, it is highly unlikely that a “one size fits all” approach is suitable. We suggest that patient phenotyping and personalized care approaches that determine the most appropriate therapy and therapy support options should be important features of an integrated sleep-disordered breathing management strategy. Individualizing care and providing the treatment most likely to be acceptable and effective for each patient should optimize therapy and improve patient outcomes.

## Data Availability

The data are not publicly available.

## Supplementary Materials

The following are available online at https://www.mdpi.com/2077-0383/10/5/936/s1, Figure S1: Probability of continuing continuous positive airway pressure (CPAP) therapy during follow-up for chronic obstructive pulmonary disease patients with or without exacerbation-related hospitalizations, Figure S2: Heatmap with hazard ratio (HR) values for risk of therapy termination in patient subgroups with different age group (years) and comorbidity combinations, Table S1: Details of codes used for CPAP therapy reimbursement, Table S2: Pharyngoplasty/UPPP codes, Table S3: Maxillofacial surgery codes, Table S4: Tonsillectomy codes, Table S5: oral appliances codes, Table S6: Identification of hypertension, ATC codes, Table S7: Non-adherence-related reasons for termination of continuous positive airway pressure therapy.
