# Supplementary material for: CPAP Therapy Termination Rates by OSA Phenotype: A French Nationwide Database Analysis
Source: J Clin Med. 2021 Mar 1;10(5):936. doi: 10.3390/jcm10050936 (PMC7957656; doi:10.3390/jcm10050936)
Supplement: Supplementary file 1 [file jcm-10-00936-s001.pdf]

Figure S1. Probability of continuing continuous positive airway pressure (CPAP) therapy during follow-up for chronic obstructive pulmonary disease patients with or without exacerbation-related hospitalizations.

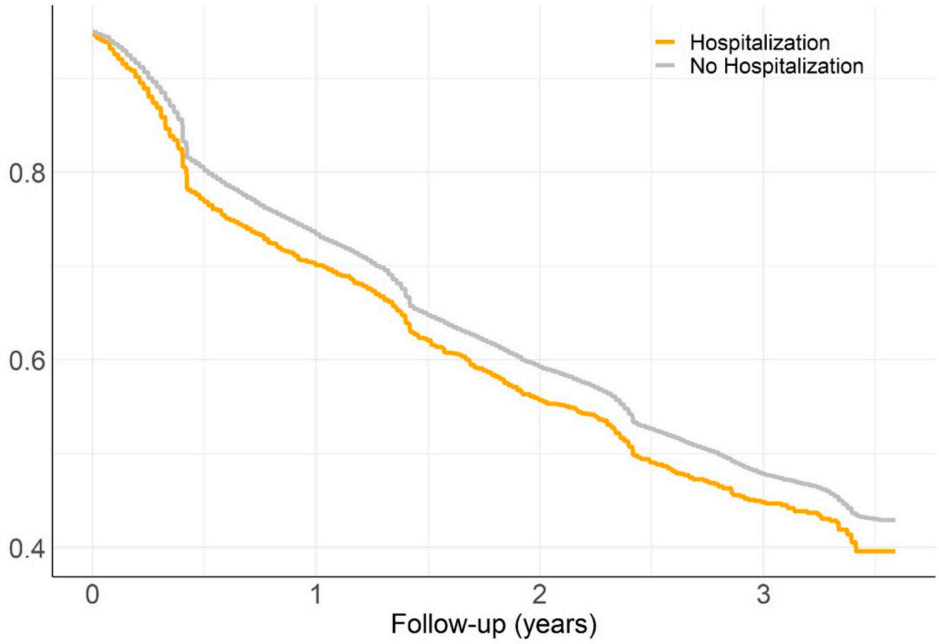

Figure S2. Heatmap with hazard ratio (HR) values for risk of therapy termination in patient subgroups with different age group (years) and comorbidity combinations. COPD, chronic obstructive pulmonary disease; HTN, hypertension. Cox model adjusted for age, sex and comorbidities. The reference patient in the multivariable model is a male patient without comorbidities aged 61-70 years. For the Cox model, the HR values are multiplicative after exponential transformation, so by multiplying the different HR values in the model it is possible to depict the HR values for specific demographic and clinical scenarios.

		Age						
		18-30	31-40	41-50	51-60	61-70	71-80	>80
**Men**	No Comorbidities	1.61	1.65	1.27	1.10	1.00	1.10	1.44
	COPD	1.80	2.07	1.59	1.38	1.26	1.38	1.81
	Diabetes	1.90	1.81	1.39	1.21	1.10	1.21	1.58
	HTN	1.55	1.57	1.21	1.05	0.95	1.04	1.37
	COPD and Diabetes	2.12	2.28	1.75	1.52	1.38	1.52	1.99
	COPD and HTN	1.73	1.97	1.51	1.32	1.20	1.31	1.72
	Diabetes and HTN	1.82	1.73	1.32	1.15	1.05	1.15	1.51
	COPD and Diabetes and HTN	2.03	2.17	1.66	1.45	1.32	1.44	1.90
**Women**	No Comorbidities	1.77	1.84	1.41	1.23	1.12	1.23	1.19
	COPD	1.97	2.32	1.78	1.54	1.40	1.54	2.02
	Diabetes	2.08	2.03	1.55	1.35	1.23	1.35	1.77
	HTN	1.70	1.75	1.35	1.17	1.06	1.17	1.53
	COPD and Diabetes	2.32	2.55	1.95	1.70	1.54	1.69	2.22
	COPD and HTN	1.89	2.21	1.69	1.47	1.34	1.47	1.93
	Diabetes and HTN	2.00	1.93	1.48	1.28	1.17	1.28	1.68
	COPD and Diabetes and HTN	2.23	2.42	1.86	1.61	1.47	1.61	2.12

Table S1. Details of codes used for CPAP therapy reimbursement

TIP code (resource : https://www.ameli.fr/etablissement/exercice-professionnel/nomenclatures-codage/lpp)
1161805, 1112830,1125301, 1108544, 1127688, 1192148, 1191864, 1122024, 1113545, 1197128, 1196962, 1182598, 1114527, 1124796, 1122337, 1142541, 1115863, 1119542, 1176340, 1149365, 1141205, 1188661, 1120723, 1103854, 1100637, 1162124, 1185361, 1157459, 1104405, 1189991, 1121131, 1137221, 1159234, 1126275, 1130897, 1126660, 1162093, 1166688, 1103156, 1188767, 1124460, 1118904, 1118614, 1133163, 1141197, 1172513, 1148064, 1169586, 1142417, 1152686, 1179248, 1129888, 1116911, 1154886, 1151250, 1102470, 1188684, 1187880, 1115455, 1192987, 1103446, 1162006, 1124112, 1106663

Codes used to identify valid explanations for CPAP therapy termination

This includes cure of sleep apnea after bariatric or otorhinolaryngology surgery, or switch to an oral appliance.

Table S2. Pharyngoplasty/UPPP codes

Code (resource: https://www.ameli.fr/etablissement/exercice-professionnel/nomenclatures-et-codage/codage-actes-medicaux-ccam)
HDMA007, HDMA009, HDMA008, HDMA005, HDMA001, HDMA004, HDMA002

Table S3. Maxillofacial surgery codes

Code (resource: https://www.ameli.fr/etablissement/exercice-professionnel/nomenclatures-et-codage/codage-actes-medicaux-ccam)
LBPA001, LBPA002, LBPA003, LBPA004, LBPA005, LBPA006, LBPA007, LBPA008, LBPA009, LBPA010, LBPA011, LBPA012, LBPA013, LBPA014, LBPA015, LBPA016, LBPA017, LBPA018, LBPA019, LBPA020, LBPA021, LBPA022, LBPA023, LBPA024, LBPA025, LBPA026, LBPA027, LBPA028, LBPA029, LBPA030, LBPA031, LBPA032, LBPA033, LBPA034, LBPA035, LBPA036, LBPA037, LBPA038, LBPA039, LBPA040, LBPA041, LBPA042, LBPA043

Table S4. Tonsillectomy codes

Code (resource: https://www.ameli.fr/etablissement/exercice-professionnel/nomenclatures-et-codage/codage-actes-medicaux-ccam)
FAFA014, FAFA015, FAFA006, FAFA005, FAFA010, FASD001, FAFA007, FAND001

Bariatric surgery

Hospitalization code : GHM 10C13

Table S5. Switch to oral appliances codes

Code (resource: https://www.ameli.fr/etablissement/exercice-professionnel/nomenclatures-codage/lpp)
2497884, 2451474, 2455325, 2465967, 2462680, 2407378, 2412971

Diabetes identification

ICD-10 code for severe and costly chronic diseases (affection de longue durée [ALD]) reimbursed at a 100% rate in France and ICD-10 codes for diabetes:

- E10 (Diabète sucré insulino–dépendant)
- E11 (Diabète sucré non insulino–dépendant)
- E12 (Diabète sucré de malnutrition)
- E13 (Autres diabètes sucrés précisés)
- E14 (Diabète sucré, sans précision)

OR

Hospitalisations as a result of diabetes-related complications were also identified using ICD-10 codes:

- E10 (Diabète sucré insulino–dépendant)
- E11 (Diabète sucré non insulino–dépendant
- E12 (Diabète sucré de malnutrition)
- E13 (Autres diabètes sucrés précisés)
- E14 (Diabète sucré, sans précision)
- G59.0* (Mononévrite diabétique)
- G63.2* (Polynévrite diabétique)
- G73.0* (Syndrome myasthénique au cours de maladie endocrinienne)
- G99.0*(Neuropathie du système nerveux autonome au cours maladies endocriniennes et métaboliques)
- H28.0* (Cataracte diabétique)
- H36.0* (Rétinopathie diabétique)

- I79.2* (Angiopathie périphérique au cours de maladies classées ailleurs)
- L97 (ulcère du membre inférieur, non classé ailleurs)
- M14.2* (Arthropathie diabétique)
- M14.6* (Arthropathie nerveuse)
- N08.3* (Glomérulopathie au cours du diabète sucré).

OR

Searching consumption of specific comorbidity-related medications using codes ATC2 "A10" (except code ATC A10BX06) delivered by both private and hospital pharmacies at least three times in the year preceding CPAP initiation.

Identification of COPD

Hospitalizations with the following codes :

- J41
- J42
- J44
- J96.1
- J43
- J44

OR

ICD-10 code for severe and costly chronic diseases (affection de longue durée [ALD]) reimbursed at a 100% rate in France and ICD-10 codes for COPD:

- J41
- J42
- J44
- J96.1

Excluding ICD-10 code for severe and costly chronic diseases (affection de longue durée [ALD]) reimbursed at a 100% rate in France and ICD-10 codes for Asthma

- J45
- J46

OR

Searching consumption of specific comorbidity-related medications using codes ATC R03 delivered by both private and hospital pharmacies at least three times in the year preceding CPAP initiation; excluding classes R03DC and R03BB04 or R03BB54)

Table S6 Identification of hypertension ATC codes

ATC codes: (resource: https://www.whocc.no/atc_ddd_index/)
C02AB02, C02AC01, C02AC02, C02AC05, C02AC06, C02CA01, C02CA06, C02DC01, C02LA01, C03AA01, C03AA03, C03BA04, C03BA10, C03BA11, C03BX03, C03CA01, C03CA02, C03CA03, C03DA01, C03DB01, C03EA, C03EA01, C03EA04, C07AA02, C07AA03, C07AA05, C07AA06, C07AA12, C07AA15, C07AA16, C07AA23, C07AB02, C07AB03, C07AB04, C07AB05, C07AB07, C07AB08, C07AB12, C07AG01, C07BA02, C07BB02, C07BB03, C07BB07, C07BB12, C07CA03, C07DA06, C07FB02, C07FB03, C08CA01, C08CA02, C08CA03, C08CA04, C08CA05, C08CA08, C08CA09, C08CA11, C08CA13, C08CX01, C08DA01, C08DB01, C08GA02, C09AA01, C09AA02, C09AA03, C09AA04, C09AA05, C09AA06, C09AA07, C09AA08, C09AA09, C09AA10, C09AA13, C09AA15, C09AA16, C09BA01, C09BA02, C09BA03, C09BA04, C09BA05, C09BA06, C09BA07, C09BA09, C09BA15, C09BB02, C09BB04, C09BB10, C09BX02, C09CA01, C09CA02, C09CA03, C09CA04, C09CA06, C09CA07, C09CA08, C09DA01, C09DA02, C09DA03, C09DA04, C09DA06, C09DA07, C09DA08, C09DB01, C09DB02, C09DB04, C09XA02, C09XA52, C10BX03

Searching consumption of specific comorbidity-related medications using following codes, delivered by both private and hospital pharmacies at least three times (separate date) in the year preceding CPAP initiation.

Table S7. Non-adherence-related reasons for termination of continuous positive airway pressure therapy

	N	%
Shift to oral appliance	736	0.19
Otorhinolaryngology surgery	172	0.04
Bariatric surgery	5,541	1.42
Death	1,731	0.44
Palliative care	12	0.00
